# Changes in taxonomic and functional diversity of plants in a chronosequence of *Eucalyptus grandis* plantations

**DOI:** 10.1038/s41598-021-89988-6

**Published:** 2021-05-24

**Authors:** Pamela E. Pairo, Estela E. Rodriguez, M. Isabel Bellocq, Pablo G. Aceñolaza

**Affiliations:** 1 Centro de Investigaciones Científicas y Transferencia de Tecnología a la Producción (CICyTTP-CONICET), Materi y España, 3105 Diamante, Entre Ríos Argentina; 2 Centro Regional de Geomática, Universidad Autónoma de Entre Ríos (CEREGEO-UADER), Ruta 11 km 10.5, 3101 Oro Verde, Entre Ríos Argentina; 3 Laboratorio de Ecología de Comunidades y Macroecología, Departamento de Ecología, Facultad de Ciencias Exactas y Naturales, Genética y Evolución, IEGEBA, (CONICET-UBA), Universidad de Buenos Aires, Ciudad Universitaria, Pabellón 2, Piso 4, 1428 Buenos Aires, CA Argentina; 4 Facultad de Ciencias Agropecuarias (FCA-UNER), Ruta 11 Km 10, 3101 Oro Verde, Entre Ríos Argentina

**Keywords:** Ecology, Environmental sciences

## Abstract

Tree plantations have become one of the fastest-growing land uses and their impact on biodiversity was evaluated mainly at the taxonomic level. The aim of this study was to analyze environmental changes after the *Eucalyptus* plantation in an area originally covered by natural grasslands, taking into account the alpha and beta (taxonomic and functional) diversity of plant communities. We selected nine plantation ages, along a 12 years chronosequence, with three replicates per age and three protected grasslands as the original situation. At each replicate, we established three plots to measure plant species cover, diversity and environmental variables. Results showed that species richness, and all diversity indices, significantly declined with increasing plantation age. Canopy cover, soil pH, and leaf litter were the environmental drivers that drove the decrease in taxonomic and functional diversity of plants through the forest chronosequence. Based on the path analyses results, canopy cover had an indirect effect on plant functional diversity, mediated by leaf litter depth, soil pH, and plant species richness. The high dispersal potential, annual, barochorous, and zoochorous plant species were the functional traits more affected by the eucalypt plantations. We recommend two management practices: reducing forest densities to allow higher light input to the understory and, due to the fact that leaf litter was negatively associated with all diversity facets, we recommend reducing their accumulation or generate heterogeneity in its distribution to enhance biodiversity.

## Introduction

The human population is growing more rapidly than ever before and consequently increasing the demand for goods and services provided by ecosystems processes^1,2^. Among human activities that imply intensive land use, tree plantations with non-native species are expanding worldwide because of the global demand for timber, pulp and biofuel products^3^. The effects of land conversion on ecosystem services will largely depend on how native biodiversity responds to the resulting environmental changes. Some studies revealed that human land uses that preserve vegetation structure and composition of natural biomes are more used by native species than those anthropogenic habitats that cause drastic environmental changes^4,5^. Specifically, tree plantations have more structural similarities to natural forests than crop fields, then promoting suitability for forest biodiversity but less than native forest^6^. In addition, studies on biodiversity changes among different plantation ages showed that the biome where the tree plantations are developed is also relevant^7^. Mature plantations contributed to maintain biodiversity better than young plantations in forest biome^8^. The opposite pattern was observed for bird and ant communities in tree plantations developed in grassland sites, where the richness and abundance were the lowest in mature plantations and highest in the grasslands^4,5,8,9^.

Several studies have already underlined the negative effects on native species and their ecosystem functions due to the expansion of forest plantations^10^. Conversely, others have pointed out that non-native tree species can restore degraded ecosystems faster than native tree plantations and have the potential to enhance above-ground biomass accumulation^11^. Plant richness, species diversity, and heterogeneity increased significantly during succession in abandoned croplands of the Loess Plateau in China^12^. However, there is evidence that that non-native plantations can affect biodiversity at different trophic levels. Zhu et al.^13^ found that the stand type (native and non-native tree plantations) had no significant effect on macroinvertebrate richness, but their abundances showed contrasting responses. The authors mentioned that such differences may be related to the microhabitat preferences of different taxa. In fact, the growth of tree plantations can alter both microclimatic conditions, soil properties and vegetation characteristics causing the process of colonization and extinction of populations within the plantations as described by the secondary succession^14^. At local scale, the suitability of tree plantations to support native biota depends on several aspects, including the planted tree species, forest density, stand age and silvicultural management^15^. Nevertheless, the influence of environmental factors on species biodiversity patterns along the forest cycle remains unresolved.

Eucalypt is one of the most common tree-planted around the world. It has been introduced in pampean grasslands replacing areas of pastures and crops altering nutrient cycles and abiotic conditions^9,16^. It has been reported that eucalypt plantations produce nutrient poor litter resulting in low soil nutrient accumulation after decomposition^17^. Such changes to soil properties affect the structure and composition of soil biological communities and, consequently, impacts on nutrient cycling processes^18^. Although the allelopathic effects and shading with the increasing canopy cover are the abiotic factors reported why eucalypt plantations reduce understory vegetation and mainly native plant species^17,19^, little is known about the link with functional diversity. Since the beginning of the twenty-first century, many ecologists have used the functional trait-based approach to complement the traditional taxonomic approach in studies of community richness and composition^20,21^. Functional traits are the characteristics of a given organism related to its response to the environment and/or its role in ecosystem functioning (Díaz and Cabido 2001) (e.g. specific leaf area, whether plants are nitrogen-fixing, dispersal mode). Thus, assessing the consequences of environmental disturbances on biological communities and ecosystem through changes in functional diversity (i.e. the value and range of functional trait values present in a community^20^), is critical to identify species traits that can dictate how species respond to environmental changes and to determine their effect on ecosystem function^22,23^.

Traditional beta diversity was defined as the variation in species composition^24^, and their study attempt to reveal the assembly mechanisms that drive the variation. More recently, functional beta diversity has been defined as the change in ecological functions or species traits between assemblages^25^. The inclusion of both facets of beta diversity is useful for understanding how human activities impact on biodiversity. Using this concept, Vaccaro et al.^26^ have shown that bird composition and functional diversity in cattle pastures, were the most similar to native pampean grasslands than other human land uses. However, the effects of tree plantations on the functional diversity of understory vegetation during the forest cycle developing in grasslands are still unclear, especially considering that they are among the most threatened and less conserved habitats^27^. Plants are often used as biodiversity indicators because they are sensitive to abiotic conditions and land-use changes, are at the base of food webs, and provide habitat for animals influencing animal diversity and distribution^28^.

The study aims to analyze the effects of the *Eucalyptus* plantation chronosequence, on alpha, beta, taxonomic and functional diversity of understory plant communities. Recognizing the structural and microclimatic changes that occur during the eucalypt forest cycle, we hypothesized that the plant taxonomic and functional similarity with the predecessor natural habitat (Pampean grasslands), will decline with decreasing environmental similarity along the plantation cycle chronosequence. Therefore, we expect that (1) as plantation age increases, environmental conditions typical of grasslands will change through the forest cycle (e.g. increase in vegetation stratification, leaf litter depth, canopy cover, and decrease in soil pH); (2) species richness and alpha functional diversity will be higher in young plantations than mature plantations; and (3) plant taxonomic and functional similarity (the inverse of beta diversity) between forest plantation and native grassland, will be higher in young plantations due to the higher environmental similarity. Given that the capability of surviving in disturbed habitats is related with life-history traits (dispersion, establishment, and persistence), our predictions about functional traits are that (1) plant communities in young plantations will have similar functional characteristics to pioneer species e.g. high potential dispersal and shorter lifespan (2) loss of functional traits typical of grassland plant species will occur as plantation age increases and (3) late-successional species may persist at the end of the forest cycle^29,30^.

## Results

We recorded a total of 247 plant species/morphospecies along the eucalypt forest cycle and grassland sites in the Mesopotamic Pampa (30 sites in total). We identified 47 species/morphospecies in grassland sites, 32 species/morphospecies in both grassland and eucalypt plantations and 168 species/morphospecies only in eucalypt plantations (Appendix A). Moreover, we identified 49 families and the most dominant were Poacea (47 spp.), Compositae (47 spp.) and Leguminosae (16 spp.).

**Figure 1:**
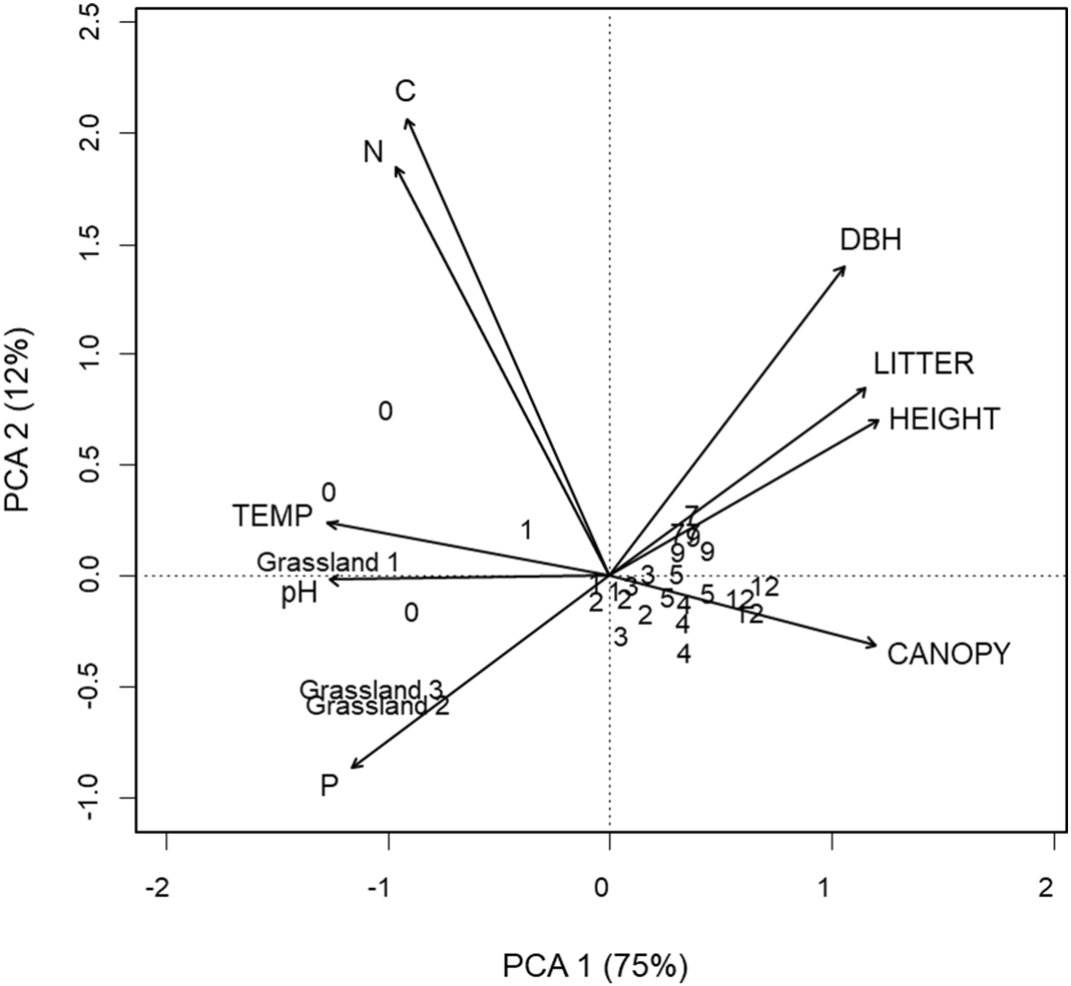
Biplot from principal component analyses (PCA) of environmental variables along eucalypt forest cycles and grassland sites. The first and second axes of the PCA are shown. The percentage of variance explained by each axis is given into brackets. CANOPY = canopy cover, LITTER = leaf litter depth, HEIGHT = eucalypt tree height, DBH = diameter at breast height, P = phosphorus content in soil, C = carbon content in soil, N = nitrogen content in soil, TEMP = temperature. Numbers represent the plantation ages.

Regarding environmental changes, the first two axes of PCA analyses explained 87% of the variation (Fig. 1). PCA axis 1 arranged sites according to plantation age from negative to positive values. Grassland sites, 0-year-old and 1-year-old plantations have similar environmental conditions: high levels of soil pH, phosphorus content and temperature. Moreover, the rest of the plantations were high canopy cover, DBH and leaf litter depth.

**Figure 2:**
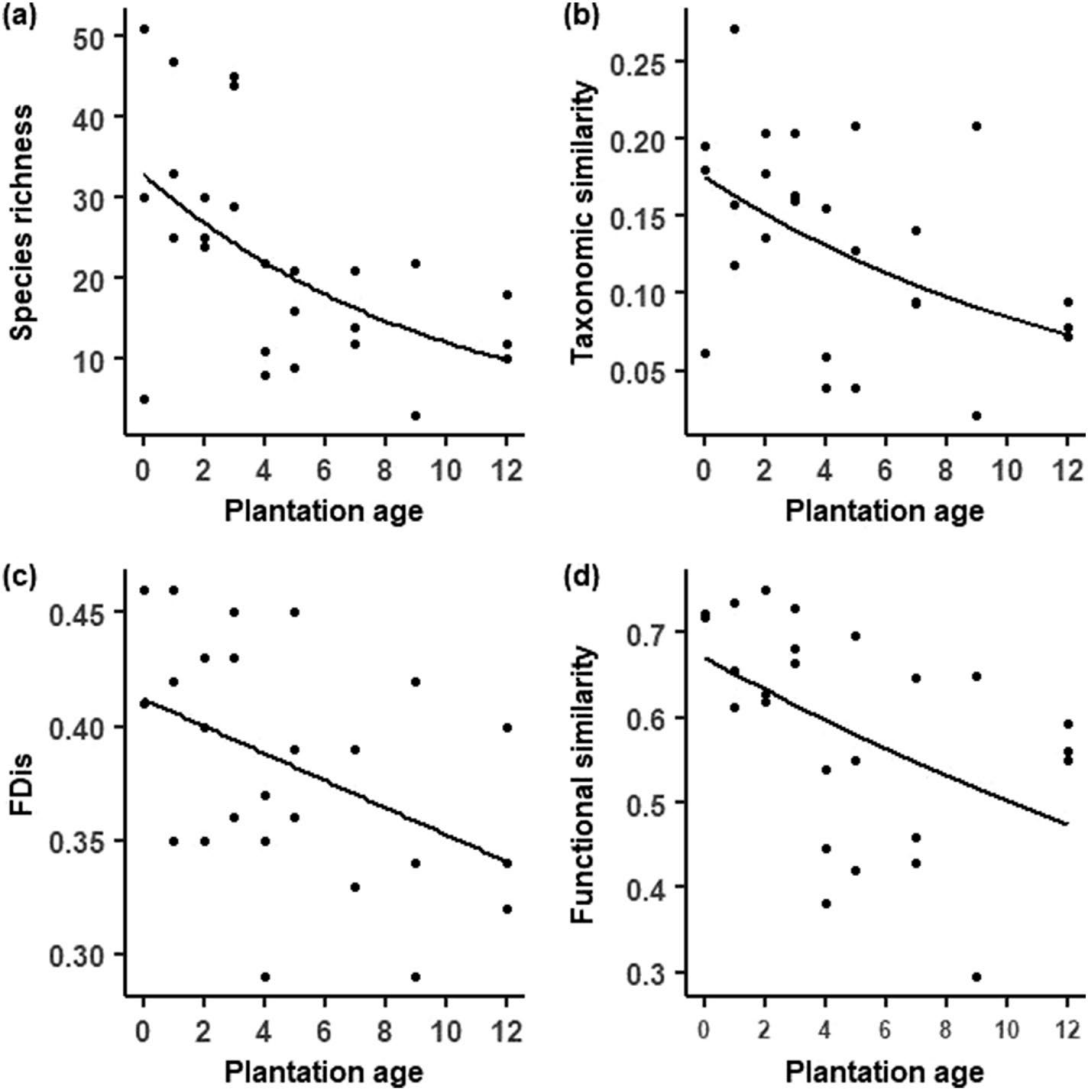
Changes of plant diversity facets along eucalypt forest cycle in grassland context. (**a**) Species richness (Z =  − 3.49, *p* < 0.001) (**b**) taxonomic similarity (1-βSor, Z =  − 2.689 *p* = 0.007) (**c**) alpha functional diversity (FDis, T =  − 2.58 *p* = 0.02) (**d**) functional similarity (1-FSor, Z =  − 2.74 *p* = 0.01). Grassland sites were pooled for measuring similarity indexes.

Plant species richness and taxonomic similarity significantly decreased as plantation age increased (Z =  − 3.49, *p* < 0.001; Z =  − 2.689, *p* = 0.007, respectively) (Fig. 2A,B). In accordance, the observed alpha functional diversity and functional similarity declined as plantation age increased (T =  − 2.58, *p* = 0.02; Z =  − 2.74, *p* = 0.01, respectively) (Fig. 2C,D).

Our results showed that plant species richness was negatively affected by soil pH, leaf litter depth, and nitrogen content (Table 1). In addition, we found that overall taxonomic similarity was also explained by similarity in leaf litter depth and canopy cover between grassland sites and the eucalypt forest plantations. The decrease of alpha functional diversity was affected by the increase of canopy cover and leaf litter depth. Finally, the decrease in functional similarity was explained by the increase of similarity in leaf litter depth, soil pH, and nitrogen content.

**Table 1:** Summary of different models that variation of diversity indices in eucalypt chronosequence in the Mesopotamic Pampa.

Diversity	N/sN	Canopy/sCanopy	Soil pH	Litter/ sLitter	d.f	AICc	%E.V
Species richness	–	–	− 0.48	− 0.15	4	207.17	41
	2.68	–	− 0.59	− 0.15	5	208.07	45
	–	–	–	− 0.12	4	209.07	30
Taxonomic similarity	−	−	−	0.75	3	− 68.27	15
	–	− 0.47	–	1.02	4	− 67.31	18
FDis (Alpha functional diversity)	–	− 0.001	–	–	3	− 87.37	20
	–	− 0.001	–	− 0.003	4	− 85.93	22
Functional similarity	–	–	–	0.41	3	− 34.40	22
	0.29	–	− 0.56	0.35	5	− 33.27	41
	–	–	− 0.33	0.35	4	− 33.19	29
	0.15	–	–	0.41	4	− 32.55	26

Best models (with ΔAICc < 2) are considered those that present similar statistical support. d.f.: degree of freedom, AICc, %E.V. = percent of explained variability of each model; pseudo R^2^ was used for beta taxonomic and functional diversity, and adjusted R^2^ for alpha functional diversity. For GLM with negative binomial distribution, we calculated the explained variability of each model as the ratio: (null deviance-residual deviance)/null deviance. sN = similarity in nitrogen content, sCanopy = similarity in canopy cover, sLitter = similarity in leaf litter depth.

**Figure 3:**
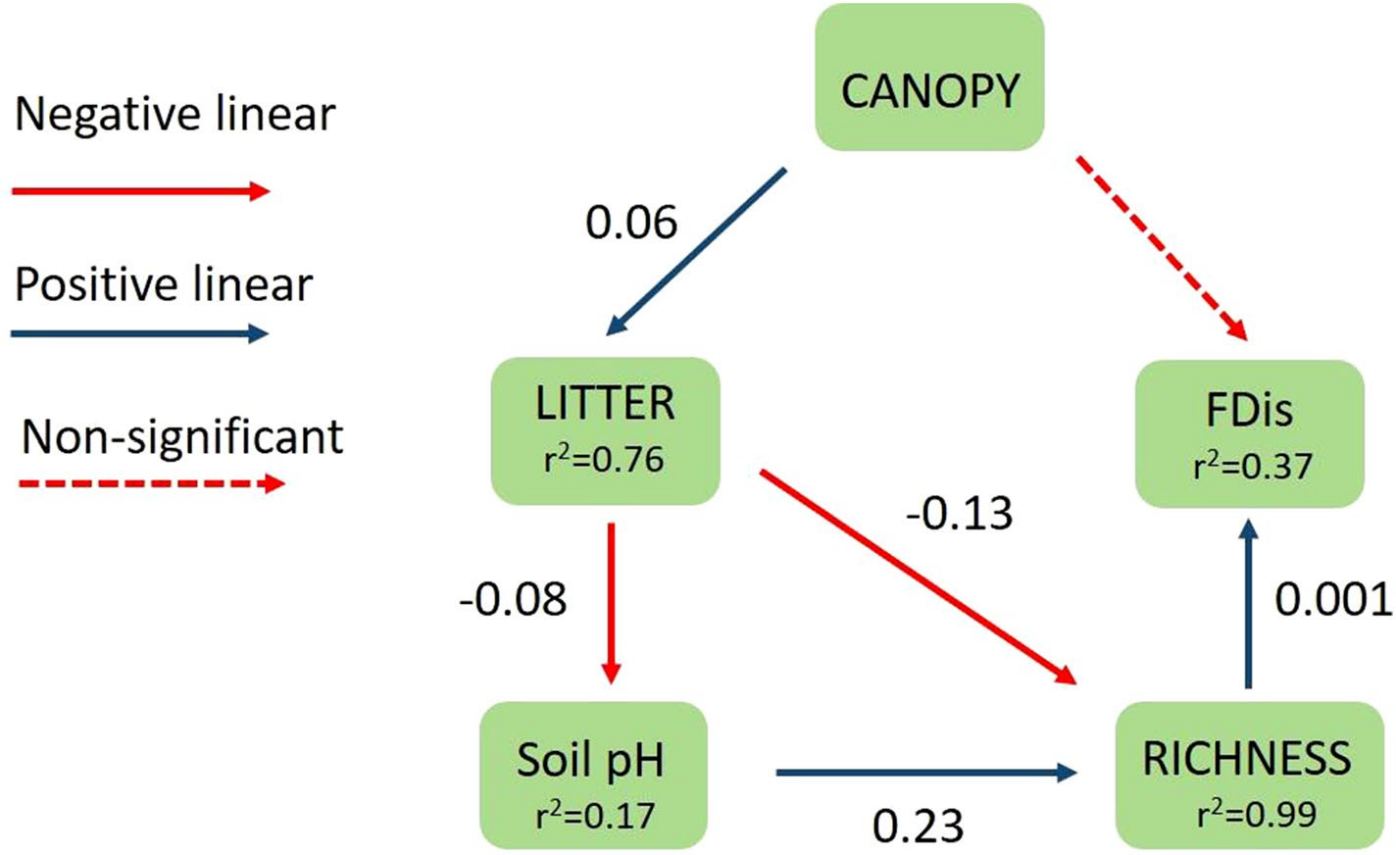
Piecewise SEM model showing the direct and indirect effect of CANOPY (canopy cover), LITTER (leaf litter depth), soil pH and RICHNESS (plant species richness) on alpha functional diversity (FDis). Arrows represent unidirectional relationships among variables and their standardized coefficients are shown (*p* < 0.05). Dashed arrow represents nonsignificant relationship (*p* > 0.05). R^2^ for component models are given in the boxes of response variables.

Piecewise SEM analysis revealed that plant species richness affected positive and directly on alpha functional diversity (Fig. 3). In addition, the model showed that the effect of the canopy cover on plant species richness is mediated by leaf litter depth and soil pH. Leaf litter depth had a direct and indirect influence on plant species richness. The analysis reproduced the data well based on a comparison of the Fisher’s C statistic to a chi-square distribution (C_8_ = 5.56, *p* = 0.7).

**Figure 4:**
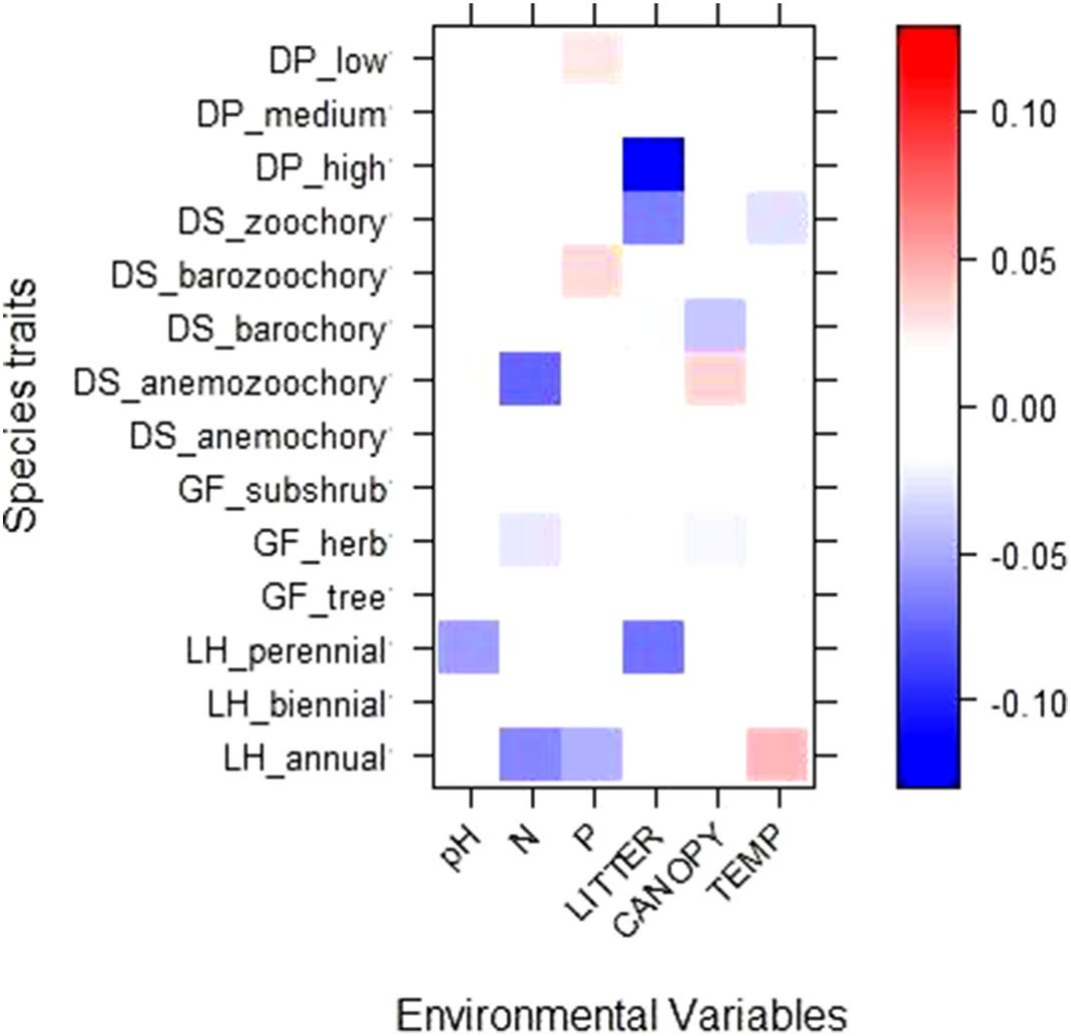
Results of fourth-corner modelling for plant trait interactions with environmental changes that occur along eucalypt forest cycle. Red represents a positive association and blue a negative association. Color grading show the direction and strengths of standardized coefficients of fourth-corner models for all environment/trait interaction terms from GLM-LASSO modelling. LH = life history, GF = growth form, DS = dispersal syndrome, DP = dispersal potential. CANOPY = canopy cover, LITTER = leaf litter depth, P = phosphorus content in soil, C = carbon content in soil, N = nitrogen content in soil, TEMP = temperature.

The fourth corner analysis revealed a positive and strong association between canopy cover and anemozoochory as dispersion syndrome and, a negative and strong association with barochorous species (Fig. 4). In addition, leaf litter depth had a negative and strong association with plants with high dispersal potential, perennial plants and zoochory and barochory as dispersal syndromes. Soil pH had a negative association with perennial plants and herbs whereas N had a negative and strong association with annual plants and anemozoochory. Finally, nitrogen content in soil had a negative strong association with annual plants and anemozoochory as dispersal syndrome.

## Discussion

Our findings reveal that changes in soil pH, canopy cover, nitrogen content in soil, and leaf litter from native grassland to mature plantations were the major drivers of the decreasing taxonomic and functional diversity of plants. To our knowledge, this is the first time to simultaneously compare plant taxonomic and functional diversities and their relationship with environmental variables providing new insights into the effects of plantation cycle on grassland plant communities. Furthermore, we propose a model that includes soil (pH) and structural variables (litter depth, canopy cover) to explain the variation in plant taxonomic and functional diversity along the eucalypt forest cycle. Eucalypt tree growth imposed a novel abiotic conditions to pampean grasslands and could modify resource availability to plant species (light, water, and nutrients)^31^.

As expected, species richness and alpha functional diversity decreased over plantation age, and it was explained by soil acidification, increasing leaf litter depth, nitrogen content in soil and increasing canopy cover along the eucalypt forest cycle. Decreasing in understory plant richness in the first four years after eucalypt plantation establishment was found within the Danling region in China where the landscape mosaic is composed of commercial plantations, cultivated land and unmanaged forests^32^. Contrary to our results, the richness and abundance of plant communities was significantly greater in mature plantations of *Robinia pseudoacacia* (non-native tree) than in the native tree plantations in the Loess Plateau in China^13^. The management practices that characterized the tree plantations caused different levels of disturbance affecting the plant community composition during the secondary succession. In eucalypt plantations, the accumulation of leaf litter has been considered a factor of allelopathy as a response to competition, through a reduction in seed germination and light availability for understory species seedling^33^. Certain phenolic acids and volatile oils are released from the leaves, bark and roots of some *Eucalyptus* spp. act as allelopathic agents and are harmful to other plant species^34^. Jobbágy et al.^35^ found that eucalypt plantations in grasslands acidify the soil mainly as a result of the release of phenolic acids. Soil acidification can also change the bioavailability of nutrients, being beneficial for certain types of species, as well as detrimental to others^36^. On the other hand, canopy cover was a relevant factor in decreasing plant functional diversity and consequently may impact on functions that plant species performed. Furthermore, light availability on the forest floor, microclimatic conditions (i.e. temperature and humidity) and rainfall patterns are closely dependent on canopy cover^37,38^. The forest canopy generate a rainfall partitioning into interception, throughfall and stemflow and then affect soil moisture patterns, infiltration, groundwater recharge and water yield^39^. Considering that high nitrogen availability in the soil could stimulate microbial activity and then an increase of decomposition rate^40^, the positive association between plant richness and nitrogen content in soil would be related to the high microbial activity allowing that certain soil nutrients are available. Therefore, the eucalypt forest cycle modified habitat characteristics limiting the establishment of some plant species and their functional traits as their ecological niches were not more compatible with the new environmental conditions.

Regarding taxonomic and functional similarity results, canopy cover, leaf litter depth, soil pH, and nitrogen content in soil were the most important factors which negatively affect grassland plant species. As we have reported previously, leaf litter could produce soil acidification and then allelopathic effects on plant species and would avoid that typical grassland species could remain. The presence of some plant species in eucalypt plantation and their absence in grassland sites (Appendix A) could be explained by the dispersion of some species from riparian forests of the Uruguay River^41^ to mature plantations. For example, we have recorded the presence of zoochory species like *Blepharocalyx salicifolius, Butia yatay, Teucrium vesicarium*and *Eugenia myrcianthes* in mature plantations*,* whose dispersion would be favored by high similarity in habitat structure and microclimatic conditions with riparian forest^8^. Besides, studies have reported that tree plantations are hot-spots of plant invasion and threaten the remnants of semi-natural vegetation^42,43^. In addition, studies that evaluated changes in plant species composition developed in landscapes dominated by forest have shown that, the species composition changes considerably due to the increasing of invasive species in commercial plantation^44–48^. On the other hand, other important factors that could influence the variability in functional diversity are management practices carried out in eucalypt plantations of pampean grasslands. Particularly forest management that invokes increased light availability through the opening spaces, for instance thinning, may effectively increase overall plant species and so biodiversity at different scales^42,49–51^. Nevertheless, the authors have remarked that this increase would be consistent with the increase of invasive plant species within tree plantations, then the original native ecosystem functions may have been damaged. Our findings suggested that thinning (in 2-year and 7-year eucalypt plantation) not increase plant diversity due to all diversity indexes showed a marked decrease in their values.

The proposed model explaining the effect of the eucalypt forest cycle on alpha functional diversity indicated that canopy cover had an indirect relationship mediated by leaf litter, soil pH, and species richness. Furthermore, the model has shown that the species richness decline was linked with the loss of unique functional traits suggesting low functional redundancy in grassland plant communities of Mesopotamic Pampa. The increase of leaf litter depth had a direct and indirect relationship with species richness would be due likely to the soil acidification and physical restrictions for plant growth. Based on the coefficients of the model, the impact caused by the physical restriction on species richness (and functional diversity) would be higher than the soil acidification and the allelopathic effects. Future studies may incorporate other abiotic factors such as nitrogen in the soil available to plants and humidity, to disentangle the effects of environmental changes on functional diversity.

The associations between life-history traits and environmental variables showed that high dispersal potential, annual, barochorous and zoochorous species were the functional traits further affected by the growth of eucalypt plantations. Furthermore, leaf litter, canopy cover, pH and nitrogen content in soil were the environmental variables with a large impact on life-history traits that could act as ecological filters and promoting that, only species with certain combinations of life traits, may pass through the filters. In addition, species that occurred in young plantations had similar characteristics to fast-growing pioneer species common in early-successional stages: high amount of seed production, annual lifeform that support our hypothesis. This concurs with the general pattern that greater dispersal ability allows quicker response following a disturbance^52^. Moreover, the increase of leaf litter depth could be a filter for grassland species which are characterized by high dispersal potential. On the other hand, zoochory and barozoochory were the dispersal syndromes affected by canopy cover and leaf litter and may impact on ecosystem services. However, we expected that anemochorous and anemozoochorus species would have been presented in young plantations, not only for their high dispersal abilities, but also as they are characteristics of grassland and can tolerate their environmental conditions. Our results are consistent with other studies that found plant traits positively associated with disturbance intensity^53^. Assemblages after disturbance comprised few plants with wind-dispersed seed, consistent with selection for species with better dispersal ability. The observed trait composition could be the response to high disturbance intensity (i.e. formicide and glyphosate application) in the early years of eucalypt plantation. This set of traits was more likely to colonize new environments and have better abilities to find the necessary resources (Duncan et al. 2003). Changes in trait composition towards generalist species in response to land-use intensification have also been reported for other traits and arthropod taxa in grasslands of Germany^54,55^. At the end of the eucalypt forest cycle, the plant species presented traits related to late-successional species (less dispersal abilities, perennial lifeform) and different dispersal syndrome with respect to younger plantations. It is important to notice that successional processes are driving plant community changes, not only the environmental changes caused by the growth of eucalypt plantations, and our study design cannot separate both effects.

Assessing the changes in taxonomic and functional diversity of plant species along the eucalypt forest cycle highlighted the importance of integrating approaches. Our results demonstrated that facets and components of native grassland plant diversity were negatively affected by novel environmental conditions provided by eucalypt plantation. Leaf litter depth was the most important driver in plant diversity decline may cause loss of native species in pampean grasslands and consequently could affect resource availability for herbivores, species interactions, trophic networks and ecosystem functioning. Our findings could be useful in places where eucalypt plantation is expanding within open habitats, especially in South America and Africa. We recommend to regional stakeholders that conservation efforts should be addressed to reduce litter accumulation and/or creating heterogeneity in its distribution within eucalypt plantations. Finally, our approaches and results may contribute to develop practical management guidelines aimed at enhancing the value of plantations for biodiversity conservation.

## Material and methods

### Study area and forest management

**Figure 5:**
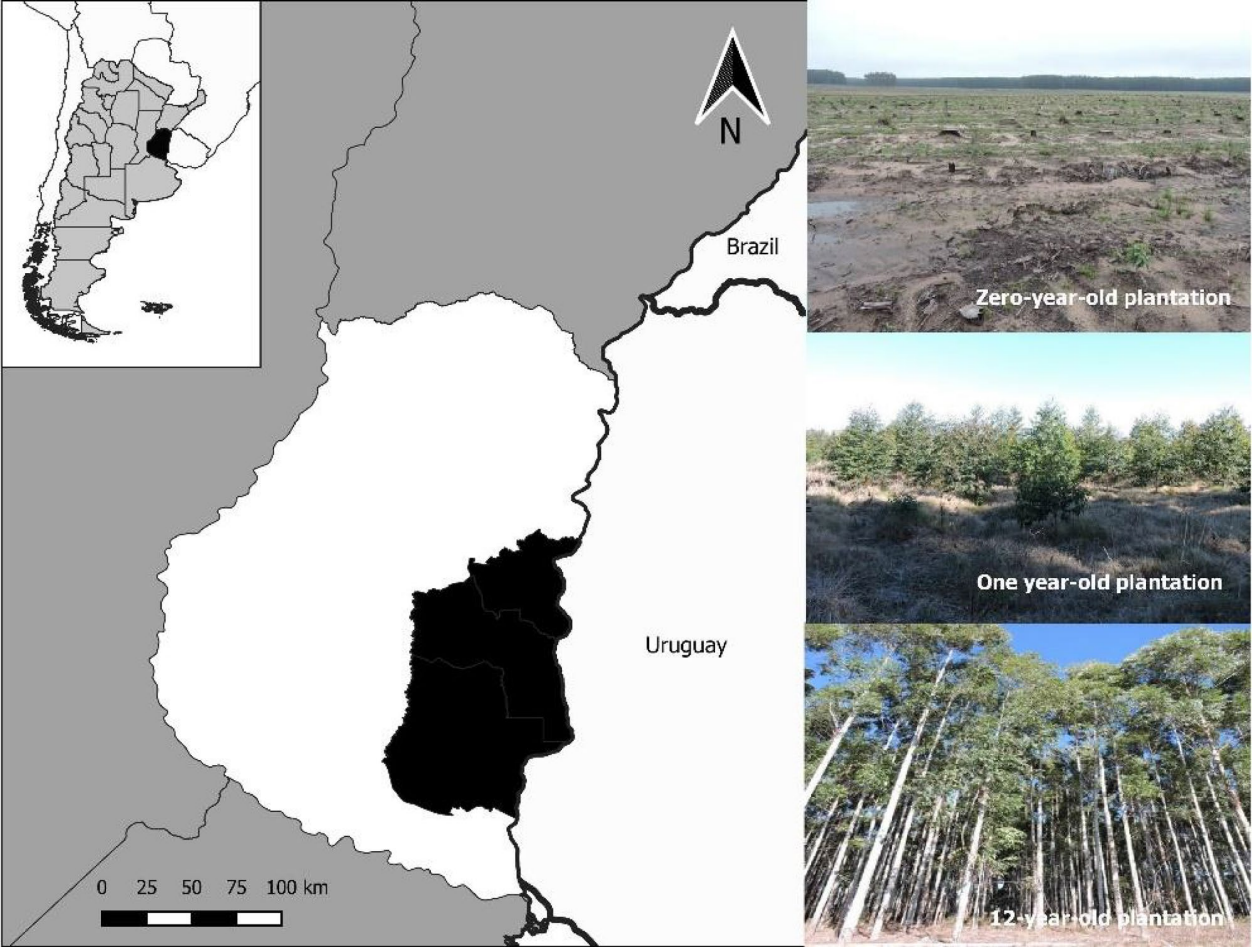
Location of the study area in the pampean grasslands in Argentina. The map was built with Quantum Geographic Information system (QGis 3.10)^88^.

The study was conducted in the Entre Ríos Province that includes the northern portion of the Pampa region (the Mesopotamic Pampa subregion), Argentina (Fig. 5). Climate is temperate humid, with an average annual temperature of 18 °C and annual average precipitation ranging from 1000 to 1200 mm^56^. The study area presents a rolling relief, well-defined streams bordered by riparian forests (Cabrera 1971; Rodriguez et al. 2017, 2018). The natural dominant vegetation type was a grassland composed of *Axonopus* sp., *Paspalum* sp., *Digitaria* sp., *Schizachyrium* sp., and *Bothriochloa* sp. (Bilenca and Miñarro 2004). The general history of anthropogenic land use in the region began with an historic extensive cattle ranching since the nineteenth century, followed by soybean cultivation and citrus crops in the twentieth century^60^. In the last 50 years, the expansion of eucalypt plantations in the Mesopotamic Pampa is replacing mainly cattle pastures, and native grassland fragments.

The Pampas region in South America is one of the greatest temperate grassland of the world and harbored one of the fastest growth planted forests in the American continent^59^. *Eucalypt grandis* W.Hill ex Maiden is the primary species planted in the region, promoted by high biomass yield, public incentives, and an emerging market of carbon sequestration^9,35^.

Specific *Eucalytus* plantations were selected with the objective of reducing the error associated with environmental variables and management practices that do not help to focus on answering the questions posed in the objectives and hypotheses. Stand selection: only plantations whose origin were natural grasslands were selected, avoiding those coming from abandoned citrus orchard or agricultural cultivation. From those, stands located in sandy loam soils and with similar site quality were filtered and finally selected. About management practices, the main criteria used in this study were, even-aged plantation; 4 × 2.5 m plantation frame which is equivalent to stocking densities averaging 1000 trees/ha^61^. The use of herbicide before planting was carried out in the planting line to reduce root competition between eucalyptus and natural grasses and herbs. In addition, formicide application was implemented to avoid herbivory from leaf-cutting ants^62,63^. Both practices were applied at 0–1 year after planting. Two pruning practice: the first one during the 3rd year, clearing all lateral branches up to 2 m height, and the second during the 6th year up to 4 m height^61^. Two thinning practices are usually conducted at 3 and 7–8 years after plantation; the final forest density is usually around 400–600 trees/ha in plantations prior to harvest^64^. Eucalypt plantations, in this area, were harvested for pulp and timber between 10 and 12 years, after that, harvest residues (leaves, bark, twigs, and branches) are not removed from stands.

### Study design, plant sampling and environmental variables

Eucalypt plantations of nine ages (0, 1, 2, 3, 4, 5, 7, 9, 12 years) were selected to represent the chronosequence; and natural grassland sites to use as reference habitat. The zero-year-old plantations referred to the period after planting but less than one year. There were three replicates per plantation age and grasslands for a total of 30 sites.

At each site, we established three 16-m^2^ plots (total 300 plots) at least 50 m apart from plantation borders to avoid edge effects. During the blooming season from the late spring to early summer (December to January), plant species were identified at each plot, assigned percentage cover and measured environmental and dasometric variables related with the forest cycle changes. The collected specimens were identified to species or morphospecies level consulting the main territorial floras^65,66^. The study complies with relevant institutional, national, and international legislation.

Moreover, canopy cover of eucalypt trees was estimated using five digital photos taken from 1.5 m above the ground toward the canopy at each site. The percent canopy on each photo was analyzed with ImageJ as percentage of pixels with vegetation^67^. The average of leaf litter depth (mostly composed by eucalypt leaves) was estimated by measuring the 10 random points per site. Similarly, average of tree height and diameter at breast height (DBH) were measured using 10 eucalypt trees. Temperature was measured with miniature temperature logger (i-button) within one replicate per age and grassland site every five min for the entire 15 days collection period. All temperature loggers were in direct contact with soil placed 10 cm above the ground level. Temperature is one of the primary factors explaining both species richness and abundances of plant likely through physiological constraints and resource availability^68^. Finally, 10 soil subsamples at 0–20 cm depth were taken by walking a ’zig-zag’ pattern from each site and mixed for homogenization. From each soil sample, total soil carbon, nitrogen, phosphorus (Bray& Kurtz), and soil pH were determined in the laboratory.

### Taxonomic diversity

To estimate alpha taxonomic diversity, we counted plant species richness per site. Moreover, to estimate beta taxonomic diversity, we calculated the Sorensen dissimilarity index (β_sor_) between each plantation age, and the native grassland pooled the three grassland sites to better represent the regional pool of species. The results of beta diversity are presented in terms of similarity in composition between communities (1-β_SOR_).

### Selection of functional traits

Plant species that occurred only once were removed from total species recorded. The main reason for this is that, dominant plant traits, are known to strongly influence ecosystem processes, indicating how the community in general responds to the environment^69^. In general, plant communities have a typical structure with a relatively small number of dominant species and a large number of subordinate or minor species that account for a low proportion of the biomass^70,71^. Plant species recorded on the plots were scored for four functional traits associated with community responses to disturbances: growth form, dispersal syndromes, dispersal potential and life history (see Table 2)^72^.

**Table 2:** Description of functional traits selected of plant species in eucalypt plantations.

Functional trait	Values	Description
Growth form	Tree, herb, subshrub	Associated with ecophysiological adaptation
Dispersal syndromes	Anemochory, anemozoochory, barochory, zoochory	Associated with the covered distances, the routes and the destination
Dispersal potential	High, medium, low	Amount of seeds produce by one individual. The capacity to survive in disturbed habitats is often correlated with a high dispersal potential
Life history	Annual, biennial, perennial	Indicator of population persistence, adult plant longevity

Information were collected from^58,65,72,87^.

### Functional diversity indices

We calculated functional dispersion (FDis) based on previous functional traits selected using the FD package^73^. FDis is defined as the mean distance in a multidimensional trait space of individual species to the centroid of all species, weighted by their relative abundances^73^. Thus, functional dispersion describes one of the facets of functional diversity, showing how far the most abundant species move away from the center of the functional space. FDis is unaffected by species richness by construction^73^.

To estimate beta functional diversity along eucalypt chronosequences (considering grassland sites as the reference habitat), we used trait matrix to build a dendrogram tree using the Jaccard index because functional traits are categorical variables and to avoid that not sharing traits increase the similarity between two species^74^. Then, both dendrogram tree and presence/absence data per site were used to calculate the Fsor index using the PICANTE package^75^, which is analogous to a traditional Sorensen’s Index. Fsor represents the proportion of dendrogram branch lengths shared by two assemblages^76^.

### Data analysis

To describe how environmental variables changed with plantation age, we carried out Principal Component Analysis (PCA) with canopy cover, soil pH, temperature, eucalypt tree height, leaf litter depth, DBH, phosphorus, carbon, and nitrogen content in soil.

To test changes in species richness (alpha taxonomic diversity) along the eucalypt forest cycle, we performed Generalized Linear Models (GLM) with Negative Binomial distribution because we had a count variable (species richness) and to reduce overdispersion. To assess changes in alpha functional diversity along the eucalypt forest cycle, we modeled FDis with Normal Distribution.

We modeled taxonomic (1 − β_SOR_) and functional similarity (Fsor) with beta distribution and performed beta regression models to explain the variability along the eucalypt forest cycle. Beta regression models are based on the assumption that the response variable is beta-distributed and it was developed for situations where, the dependent variable, can take values between 0 and 1 as the case of the Sorensen index^77^.

To identify the main environmental variables that explain changes of taxonomic diversity along the eucalypt forest cycle, we used a model-selection approach with Akaike Criterion (AICc), as we handled the corrected version for small sample sizes^78^. Models with ∆AICc < 2 were considered as equivalent to the minimum AICc model and hence more robust to explain the observed variability^79^. Goodness-of-fit was evaluated by examining plots of standardized residuals versus predicted and checked normal distribution. Environmental similarity indices (1-Gower index), between each environmental variable respect to grassland sites, were calculated for beta taxonomic analyses. The models with ΔAICc > 2 have been present in Appendix B, C, D, and E.

As noted previously for taxonomic diversity analyses, the model selection approach was implemented to identify the environmental variables that explained the variability in alpha functional diversity and functional similarity along the eucalypt plantation cycle.

Structural equation models (SEMs) are a statistical technique that unite multiple variables in a single causal network, thereby allowing simultaneous tests of multiples hypotheses^80^. SEMs test the direct and indirect effects on pre-assumed causal relationships^81^. Piecewise SEM expands upon traditional SEMs by introducing a flexible mathematical framework that can incorporate a non-normal distributions, hierarchical structures and different estimation procedures^82^. We then performed a piecewise SEM to analyze the direct and indirect effects on the alfa functional diversity (FDis), including the environmental variables with the lowest AICc from the model selection and the plant species richness. Some researchers recommend a minimum sample size of five cases per free parameter in the model resulting in six possible relationships to be tested^81^. The direct relationship between species richness and alpha functional diversity was modeled with Poisson distribution. Fisher’s C statistic was calculated to evaluate the global goodness-of-fit of the model (*P* > 0.05) using the ‘psem’ function from the ‘piecewiseSEM’ package^83^.

In order to identify associations between functional traits and environmental variables, we used fourth-corner analysis, i.e. measured through the interaction terms between each environmental variable and trait variables^84^. The sign and magnitude of the interaction coefficients denoted the nature and strength of the association of the trait–environment relationship, respectively. We fitted a linear regression model, using all functional traits and environmental variables and the LASSO penalty was estimated via cross-validation. LASSO is a method of penalized likelihood which imposes a constraint on estimates of model parameters^85^.

All statistical analyses were conducted in R 3.2.0. Beta regression was performed using functions in the R package ‘betareg’^77^, and candidate models were selected with the R package ‘MuMIn’^86^.

## Supplementary Information

Supplementary Appendix A.

Supplementary Appendix B.

Supplementary Appendix C.

Supplementary Appendix D.

Supplementary Appendix E.

Supplementary Information.

## Data Availability

The presence of plant species in eucalypt plantations and grasslands sites are in the Supplementary Information. More detail information of this study is available from the corresponding author on reasonable request.

## References

[1] Sala OE (2000). Global biodiversity scenarios for the year 2100. Science (80- ).

[2] Wall DH, Nielsen UN (2012). Biodiversity and ecosystem services: is it the same below ground?. Nat. Educ. Knowl..

[3] FAO. *Global Forest Resources Assessment 2015: Desk Reference*. http://www.fao.org/3/a-i4808e.pdf (2015).

[4] Filloy J, Zurita GA, Corbelli JM, Bellocq MI (2010). On the similarity among bird communities: testing the influence of distance and land use. Acta Oecol..

[5] Santoandré S, Filloy J, Zurita GA, Bellocq MI (2019). Ant taxonomic and functional diversity show differential response to plantation age in two contrasting biomes. For. Ecol. Manag..

[6] Calviño-Cancela M (2013). Effectiveness of eucalypt plantations as a surrogate habitat for birds. For. Ecol. Manag..

[7] Santoandré S, Filloy J, Zurita GA, Bellocq MI (2019). Taxonomic and functional β-diversity of ants along tree plantation chronosequences differ between contrasting biomes. Basic Appl. Ecol..

[8] Corbelli JM (2015). Integrating taxonomic, functional and phylogenetic beta diversities: interactive effects with the biome and land use across taxa. PLoS ONE.

[9] Phifer CC, Knowlton JL, Webster CR, Flaspohler DJ, Licata JA (2016). Bird community responses to afforested eucalyptus plantations in the Argentine pampas. Biodivers. Conserv..

[10] Tererai F, Gaertner M, Jacobs SM, Richardson DM (2013). Eucalyptus invasions in riparian forests: effects on native vegetation community diversity, stand structure and composition. For. Ecol. Manag..

[11] Brancalion PHS (2019). Intensive silviculture enhances biomass accumulation and tree diversity recovery in tropical forest restoration. Ecol. Appl..

[12] Zhang C, Liu G, Xue S, Wang G (2016). Soil bacterial community dynamics reflect changes in plant community and soil properties during the secondary succession of abandoned farmland in the Loess Plateau. Soil Biol. Biochem..

[13] Zhu Y, Wang Y, Chen L (2019). Effects of non-native tree plantations on the diversity of understory plants and soil macroinvertebrates in the Loess Plateau of China. Plant Soil.

[14] Zhang W (2018). Plant functional composition and species diversity affect soil C, N, and P during secondary succession of abandoned farmland on the Loess Plateau. Ecol. Eng..

[15] Munévar A, Rubio GD, Andrés G (2018). Changes in spider diversity through the growth cycle of pine plantations in the semi-deciduous Atlantic forest: the role of prey availability and abiotic conditions. For. Ecol. Manag..

[16] Vega E, Baldi G, Jobbágy EG, Paruelo J (2009). Land use change patterns in the Río de la Plata grasslands: the influence of phytogeographic and political boundaries. Agric. Ecosyst. Environ..

[17] Ntshuxeko VE, Ruwanza S (2018). Physical properties of soil in Pine elliottii and Eucalyptus cloeziana plantations in the Vhembe biosphere, Limpopo Province of South Africa. J. For. Res..

[18] Kerr TF, Ruwanza S (2016). Does *Eucalyptus grandis* invasion and removal affect soils and vegetation in the Eastern Cape Province, South Africa?. Austral. Ecol..

[19] Zhang DJ, Zhang J, Yang WQ, Wu FZ (2010). Potential allelopathic effect of Eucalyptus grandis across a range of plantation ages. Ecol. Res..

[20] Díaz S, Cabido M (2001). Vive la difference: plant functional diversity matters to ecosystem processes: plant functional diversity matters to ecosystem processes. Trends Ecol. Evol..

[21] Petchey OL, Gaston KJ (2006). Functional diversity: back to basics and looking forward. Ecol. Lett..

[22] Luck GW, Lavorel S, Mcintyre S, Lumb K (2012). Improving the application of vertebrate trait-based frameworks to the study of ecosystem services. J. Anim. Ecol..

[23] Lindenmayer D (2015). Richness is not all: how changes in avian functional diversity reflect major landscape modification caused by pine plantations. Divers. Distrib..

[24] Whittaker RH (1960). Vegetation of the Siskiyou Mountains, Oregon and California. Ecol. Monogr..

[25] Swenson, N. G. *Functional and Phylogenetic Ecology in R*. *Use R!* (2014). 10.1007/978-1-4614-9542-0.

[26] Vaccaro AS, Filloy J, Bellocq MI (2019). What land use better preserves taxonomic and functional diversity of birds in a grassland biome?. Avian Conserv. Ecol..

[27] Blair, J., Nippert, J. & Briggs, J. *Grassland Ecology*. *Ecology and the Environment* vol. 8 (Springer, 2014).

[28] Nic Lughadha E (2005). Measuring the fate of plant diversity: towards a foundation for future monitoring and opportunities for urgent action. Philos. Trans. R. Soc. B Biol. Sci..

[29] Marteinsdóttir B, Eriksson O (2014). Trait-based filtering from the regional species pool into local grassland communities. J. Plant Ecol..

[30] Salgado Negret, B. *La Ecología Funcional como aproximación al estudio, manejo y conservación de la biodiversidad: protocolos y aplicaciones*. *La ecología funcional como aproximación al estudio, manejo y conservación de la biodiversidad: protocolos y aplicaciones* (2015).

[31] Barbier S, Gosselin F, Balandier P (2008). Influence of tree species on understory vegetation diversity and mechanisms involved—a critical review for temperate and boreal forests. For. Ecol. Manag..

[32] Zhang D, Zhang J, Yang W, Wu F, Huang Y (2014). Plant and soil seed bank diversity across a range of ages of *Eucalyptus grandis* plantations afforested on arable lands. Plant Soil.

[33] Zhang C, Fu S (2009). Allelopathic effects of eucalyptus and the establishment of mixed stands of eucalyptus and native species. For. Ecol. Manag..

[34] Florentine SK, Fox JED (2003). Allelopathic effects of *Eucalyptus victrix* L. on Eucalyptus species and grasses. Allelopath. J..

[35] Jobbágy, E. *et al.* Forestación en pastizales: hacia una visión integral de sus oportunidades y costos ecológicos. *Agrociencia* **X**, 109–124 (2006).

[36] Ruwanza S, Gaertner M, Esler KJ, Richardson DM (2015). Allelopathic effects of invasive Eucalyptus camaldulensis on germination and early growth of four native species in the Western Cape South Africa. South. For..

[37] Suggitt AJ (2011). Habitat microclimates drive fi ne-scale variation in extreme temperatures. Oikos.

[38] Zellweger F, Roth T, Bugmann H, Bollmann K (2017). Beta diversity of plants, birds and butterflies is closely associated with climate and habitat structure. Glob. Ecol. Biogeogr..

[39] Silveira L, Alonso J (2009). Runoff modifications due to the conversion of natural grasslands to forests in a large basin in Uruguay. Hidrol. Process..

[40] Mendoza CA, Gallardo JF, Turrión MB, Pando V, Aceñolaza PG (2017). Dry weight loss in leaves of dominant species in a successional sequence of the Mesopotamian Espinal (Argentina). For. Syst..

[41] Rodriguez EE, Aceñolaza PG, Perea EL, Galán de Mera A (2017). A phytosociological analysis of Butia yatay (Arecaceae) palm groves and gallery forests in Entre Rios, Argentina. Aust. J. Bot..

[42] Piwczyński M, Puchałka R, Ulrich W (2016). Influence of tree plantations on the phylogenetic structure of understorey plant communities. For. Ecol. Manag..

[43] Csecserits A (2016). Tree plantations are hot-spots of plant invasion in a landscape with heterogeneous land-use. Agric. Ecosyst. Environ..

[44] Amazonas NT (2018). High diversity mixed plantations of Eucalyptus and native trees: an interface between production and restoration for the tropics. For. Ecol. Manag..

[45] Verstraeten G (2013). Understorey vegetation shifts following the conversion of temperate deciduous forest to spruce plantation. For. Ecol. Manag..

[46] Grass I, Brandl R, Botzat A, Neuschulz EL, Farwig N (2015). Contrasting taxonomic and phylogenetic diversity responses to forest modifications: comparisons of taxa and successive plant life stages in south African scarp forest. PLoS ONE.

[47] Wu J (2015). Should exotic Eucalyptus be planted in subtropical China: insights from understory plant diversity in two contrasting Eucalyptus chronosequences. Environ. Manag..

[48] Jin D (2016). High risk of plant invasion in the understory of eucalypt plantations in South China. Sci. Rep..

[49] Haughian SR, Frego KA (2016). Short-term effects of three commercial thinning treatments on diversity of understory vascular plants in white spruce plantations of northern New Brunswick. For. Ecol. Manag..

[50] Smith GF, Iremonger S, Kelly DL, O’Donoghue S, Mitchell FJG (2007). Enhancing vegetation diversity in glades, rides and roads in plantation forests. Biol. Conserv..

[51] Aceñolaza, P. G., Rodriguez, E. E. & Diaz, D. Efecto de prácticas de manejo silvícola sobre la diversidad vegetal bajo plantaciones de Eucalyptus grandis. In *4to Congreso Forestal Argentino y Latinoamericano* (2013).

[52] Connell JH, Slatyer RO (1977). Mechanisms of succession in natural communities and their role in community stability and organization. Am. Nat..

[53] Pedley SM, Dolman PM (2014). Multi-taxa trait and functional responses to physical disturbance. J. Anim. Ecol..

[54] Birkhofer K, Smith HG, Weisser WW, Wolters V, Gossner MM (2015). Land-use effects on the functional distinctness of arthropod communities. Ecography (Cop.).

[55] Mangels J, Fiedler K, Schneider FD, Blu N (2017). Diversity and trait composition of moths respond to land-use intensification in grasslands : generalists replace specialists. Biodivers. Conserv..

[56] Morello, J., Matteucci, S. D., Rodriguez, A. F. & Silva, M. *Ecorregiones y complejos ecosistemicos argentino*. (2012).

[57] Cabrera Á (1971). Fitogeografía de la República Argentina. Bol. Soc. Argent. Bot..

[58] Rodriguez, E. E., Aceñolaza, P. G., Picasso, G. & Gago, J. *Plantas del bajo Rio Uruguay: árboles, arbustos, herbáceas, lianas y epifitas.* (2018).

[59] Bilenca D, Miñarro F (2004). Identificación de Áreas Valiosas de Pastizal (AVPs) en las Pampas y Campos de Argentina Uruguay y sur de Brasil. Vasa.

[60] Inta. Plan de Tecnologia Regional 2009–2011. *INTA Cent. Reg. Entre Rios* (2011).

[61] Aguerre, M. *et al. Manual para productores de Eucaliptos de la Mesopotamia Argentina.* (1995).

[62] Aparicio, J. L., Larocca, F. & Dalla Tea, F. Silvicultura de establecimiento de Eucalyptus grandis. *IDIA XXI, Revista de Información sobre Investigación y Desarrollo Agropecuario* 66–69 (2005).

[63] Vilela E, Leite HG, Jaffe K (2015). Level of economic damage for leaf-cutting ants (Hymenoptera: Formicidae) in Eucalyptus plantations in Brazil. Sociobiology.

[64] Larroca, F., Dalla Tea, F. & Aparicio, J. L. Técnicas de implantación y manejo de eucaliptus para pequeños y medianos forestadores en Entre Ríos y Corrientes. in *XIX Jornadas Forestales de Entre Ríos.* (2004).

[65] Burkart, A. *Flora ilustrada de la provincia de Entre Ríos.* (INTA, 1969).

[66] Burkart, A. *Flora ilustrada de Entre Ríos (Argentina). Parte 2 Gramíneas*. *Colección Científica del INTA* (1969).

[67] Peyras M, Vespa NI, Bellocq MI, Zurita GA (2013). Quantifying edge effects : the role of habitat contrast and species specialization. J. Insect Conserv..

[68] Werenkraut V, Fergnani PN, Ruggiero A (2015). Ants at the edge: a sharp forest-steppe boundary influences the taxonomic and functional organization of ant species assemblages along elevational gradients in northwestern Patagonia (Argentina). Biodivers. Conserv..

[69] Diaz S, Cabido M, Casanoves F (1998). Plant functional traits and environmental filters at a regional scale. J. Veg. Sci..

[70] Grime JP (1998). Benefits of plant diversity to ecosystems: immediate, filter and founder effects. J. Ecol..

[71] Carreño-Rocabado G (2015). Land-use intensification effects on functional properties in tropical plant communities. Ecol. Appl..

[72] Pérez-Harguindeguy N (2013). New Handbook for standardized measurment of plant functional traits worldwide. Aust. J. Bot..

[73] Laliberté E, Legendre P (2010). A distance-based framework for measuring functional diversity from multiple traits. Ecology.

[74] Legendre, P. & Legendre, L. F. J. *Numerical Ecology*. (Elsevier, 2012).

[75] Kembel, S. W. *et al.* Package ‘ picante ’: Integrating Phylogenies and Ecology. *Cran-R* 1–55 (2018). 10.1093/bioinformatics/btq166>.License. 10.1093/bioinformatics/btq166 20395285

[76] Swenson NG, Anglada-Cordero P, Barone JA (2011). Deterministic tropical tree community turnover: evidence from patterns of functional beta diversity along an elevational gradient. Proc. R. Soc. B Biol. Sci..

[77] Cribari-Neto F, Zeileis A (2010). Journal of Statistical Software. J. Stat. Softw..

[78] Burnham KP, Anderson DR (2002). Model Selection and Multimodel Inference: A Practical Information-Theoretic Approach.

[79] Zuur AF, Ieno EN, Elphick CS (2010). A protocol for data exploration to avoid common statistical problems. Methods Ecol. Evol..

[80] Grace, J. B. *Structural Equation Modeling and Natural Systems*. (Cambridge University Press, 2006).

[81] Fan Y (2016). Applications of structural equation modeling (SEM) in ecological studies: an updated review. Ecol. Process..

[82] Lefcheck JS (2016). piecewiseSEM: piecewise structural equation modelling in r for ecology, evolution, and systematics. Methods Ecol. Evol..

[83] Lefcheck, J., Byrnes, J. & Grace, J. Package ‘ piecewiseSEM ’. *R* (2019).

[84] Brown AM (2014). The fourth-corner solution - using predictive models to understand how species traits interact with the environment. Methods Ecol. Evol..

[85] Hastie, T., Tibshirani, R. & Friedman, J. *The Elements of Statistical Learning: Data Mining, Inference and Prediction*. (2009).

[86] Barton, K. Package ‘MuMIn’.Multi-Model Inference. (2018).

[87] Dawson SK (2017). Plant traits of propagule banks and standing vegetation reveal flooding alleviates impacts of agriculture on wetland restoration. J. Appl. Ecol..

[88] QGIS Development Team. QGIS Geographic Information System. Open Source Geospatial Foundation Project. (2019). http://qgis.osgeo.org

