# Supplementary material for: Changes in taxonomic and functional diversity of plants in a chronosequence of Eucalyptus grandis plantations
Source: Sci Rep. 2021 May 24;11:10768. doi: 10.1038/s41598-021-89988-6 (PMC8144202; doi:10.1038/s41598-021-89988-6)
Supplement: Supplementary file 1 — Supplementary Appendix A. [file 41598_2021_89988_MOESM1_ESM.docx]

**Eucalyptus plantations in temperate grasslands: responses of taxonomic and functional diversity of plant communities to environmental changes**

**Pamela E. Pairo^1, 3^*, Estela E. Rodriguez^1, 2^, M. Isabel Bellocq^3^ and Pablo G. Aceñolaza^1, 2,4^**

^1^Centro de Investigaciones Científicas y Transferencia de Tecnología a la Producción (CICyTTP-CONICET), Materi y España (3105) Diamante, Entre Ríos, Argentina.

^2^Centro Regional de Geomática, Universidad Autónoma de Entre Ríos (CEREGEO-UADER), Ruta 11 km 10.5, Oro Verde (3101) Entre Ríos, Argentina.

^3^Laboratorio de Ecología de Comunidades y Macroecología, Departamento de Ecología, Genética y Evolución, IEGEBA, (CONICET-UBA), Facultad de Ciencias Exactas y Naturales, Universidad de Buenos Aires, Ciudad Universitaria, Pabellón 2, Piso 4, CA Buenos Aires (1428), Argentina.

^4^ Facultad de Ciencias Agropecuarias (FCA-UNER). Ruta 11 Km 10 (3101), Oro Verde, Entre Ríos, Argentina.

* E-mail: [pamepairo@gmail.com](mailto:pamepairo@gmail.com)

**Appendix A.** List of plant species/morphospecies recorded in grassland sites and eucalypt plantation in Mesopotamic Pampa of Entre Rios Province, Argentina. Nomenclatural and author data of plants follow The Plant List (The Plant List 2013).

| **Family** | **Plant species** | **Grassland** | **Forest plantation** |  |
| --- | --- | --- | --- | --- |
| Amaranthaceae | *Amaranthus sp.* |  | x |  |
|  | *Amaranthus hybridus subsp. quitensis*(Kunth) Costea & Carretero |  | x |  |
|  | *Amaranthus viridis L.* |  | x |  |
|  | *Froelichia interrupta*(L.) Moq. | x |  |  |
|  | *Iresine diffusa*Humb. & Bonpl. ex Willd*.* |  | x |  |
|  | *Pfaffia sp.* |  | x |  |
|  | *Pfaffia gnaphalioides*(L.f.) Mart. |  | x |  |
| Amaryllidaceae | *Nothoscordum sp.* |  | x |  |
| Apiaceae | *Cyclospermum leptophyllum (*Pers.) Sprague |  | x |  |
|  | *Daucus pusillus*Michx. | x | x |  |
|  | *Eryngium horridum* Malme. |  | x |  |
|  | *Eryngium nudicaule* Lam. |  | x |  |
|  | *Eryngium sp.* |  | x |  |
|  | *Eryngium elegans*Cham. & Schltdl*.* |  | x |  |
| Apocynaceae | *Mandevilla petraea* (A.St.-Hil.) Pichon | x |  |  |
|  | *Asclepias curassavica* L. |  | x |  |
|  | *Morrenia brachystephana*Griseb. |  | x |  |
|  | *Oxypetalum solanoides*Hook. & Arn. |  | x |  |
| Araliaceae | *Hydrocotyle sp.* |  | x |  |
| Arecaceae | *Butia yatay*(Mart.) Becc. |  | x |  |
| Aristolochiaceae | *Aristolochia sp.* |  | x |  |
| Boraginaceae | *Echium plantagineum*L. |  | x |  |
|  | *Heliotropium amplexicaule* Vahl |  | x |  |
|  | *Heliotropium nicotianifolium*Poir. |  | x |  |
| Cactaceae | *Opuntia sp.* | x |  |  |
| Calyceraceae | *Acicarpha tribuloides*Juss. | x | x |  |
| Campanulaceae | *Triodanis perfoliata subsp. biflora*(Ruiz & Pav.) Lammers |  | x |  |
|  | *Wahlenbergia linarioides*(Lam.) A. DC | x | x |  |
| Caryophyllaceae | *Paronychia communis*Cambess. |  | x |  |
|  | *Silene antirrhina*L. | x |  |  |
|  | *Silene sp.* | x |  |  |
|  | *Spergula sp.* | x |  |  |
|  | *Stellaria media*(L.) Vill. |  | x |  |
| Commelinaceae | *Commelina diffusa*Burm.f. |  | x |  |
|  | *Commelina erecta*L. | x | x |  |
|  | *Commelina platyphylla*Klotzsch ex Seub. |  | x |  |
|  | *Commelina sp.* | x |  |  |
|  | *Tripogandra glandulosa*(Seub.) Rohweder | x | x |  |
| Compositae | *Acanthostyles buniifolius*(Hook. ex Hook. & Arn.) R.M.King & H. Rob. |  | x |  |
|  | *Achyrocline satureioides*(Lam.) DC*.* | x | x |  |
|  | *Acmella grisea*(Chodat) R.K.Jansen |  | x |  |
|  | *Ambrosia tenuifolia*Spreng. |  | x |  |
|  | *Aster squamatus*(Spreng.) Hieron*.* |  | x |  |
|  | *Baccharis articulata*(Lam.) Pers. |  | x |  |
|  | *Baccharis dracunculifolia*DC. | x | x |  |
|  | *Baccharis pingraea* DC. |  | x |  |
|  | *Baccharis* *rufescens* var. *rufescens* | x |  |  |
|  | *Baccharis salicina*Torr. & A.Gray |  | x |  |
|  | *Baccharis sp.* | x | x |  |
|  | *Baccharis sp.1* |  | x |  |
|  | *Baccharis trimera*(Less.) DC. |  | x |  |
|  | *Bidens sp.* |  | x |  |
|  | *Calea uniflora*Less. | x |  |  |
|  | *Campuloclinium macrocephalum*(Less.) DC*.* |  | x |  |
|  | *Carduus acanthoides*L. |  | x |  |
|  | *Cirsium vulgare (Savi)* Ten. |  | x |  |
|  | *Erigeron bonariensis L.* | x | x |  |
|  | *Eupatorium inulifolium*Kunth |  | x |  |
|  | *Eupatorium sp.* | x |  |  |
|  | *Eupatorium sp. 1* |  | x |  |
|  | *Gnaphalium americanum*Mill. |  | x |  |
|  | *Gamochaeta argentina* Cabrera | x | x |  |
|  | *Gamochaeta simplicicaulis*(Willd. ex Spreng.) *Cabrera* | x | x |  |
|  | *Gamochaeta stagnalis*(I.M.Johnst.) Anderb. |  | x |  |
|  | *Gnaphalium filagineum* DC*.* |  | x |  |
|  | *Helminthotheca echioides*(L.) Holub | x |  |  |
|  | *Hypochaeris glabra* L. |  | x |  |
|  | *Hysterionica montevidensis*Baker |  | x |  |
|  | *Lucilia acutifolia*(Poir.) Cass | x |  |  |
|  | *Mikania cordifolia*(L.f.) Willd. |  | x |  |
|  | *Porophyllum ruderale*(Jacq.) Cass. |  | x |  |
|  | *Pseudognaphalium gaudichaudianum*(DC.) Anderb. |  | x |  |
|  | *Pterocaulon lorentzii*Malme | x | x |  |
|  | *Pterocaulon polystachyum*DC |  | x |  |
|  | *Senecio crassiflorus*(Poir.) DC. |  | x |  |
|  | *Senecio madagascariensis*Poir. |  | x |  |
|  | *Senecio selloi*(Spreng.) DC. |  | x |  |
|  | *Silybum marianum*(L.) Gaertn. |  | x |  |
|  | *Solidago chilensis*Meyen |  | x |  |
|  | *Sonchus asper*(L.) Hill |  | x |  |
|  | *Sonchus oleraceus*(L.) L. |  | x |  |
|  | *Tagetes minuta*L. |  | x |  |
|  | *Trixis pallida*Less. | x |  |  |
|  | *Vernonia incana*Less. | x | x |  |
|  | *Xanthium spinosum*L. |  | x |  |
| Convolvulaceae | *Dichondra macrocalyx*Meisn. |  | x |  |
|  | *Dichondra sericea*Sw*.* | x | x |  |
|  | *Evolvulus sericeus f. pedunculatus*Ooststr. | x | x |  |
| Cucurbitaceae | *Cayaponia sp.* |  | x |  |
| Cyperaceae | *Carex trachycystis* Griseb. |  | x |  |
|  | *Carex sp.* |  | x |  |
|  | *Cyperus sp.* | x | x |  |
|  | *Cyperus sp. 1* | x | x |  |
|  | *Cyperus sp. 2* |  | x |  |
|  | *Cyperus sp. 3* |  | x |  |
|  | *Cyperus sp. 4* |  | x |  |
|  | *Cyperus sp. 5* |  | x |  |
|  | *Fimbristylis autumnalis (L.)* Roem. & Schult. | x | x |  |
|  | *Kyllinga odorata*Vahl |  | x |  |
| Euphorbiaceae | *Acalypha communis*Müll.Arg. | x |  |  |
|  | *Astraea lobata*(L.) Klotzsch |  | x |  |
|  | *Bernardia sellowii*Müll.Arg. | x |  |  |
|  | *Croton sp.* | x |  |  |
|  | *Croton glandulosus L.* |  | x |  |
|  | *Euphorbia sp.* | x |  |  |
|  | *Euphorbia serpens*Kunth |  | x |  |
|  | *Tragia geraniifolia*Klotzsch ex Müll.Arg | x | x |  |
|  | *Tragia volubilis L.* |  | x |  |
| Gentianaceae | *Centaurium pulchellum*(Sw.) Druce |  | x |  |
| Geraniaceae | *Geranium carolinianum L.* |  | x |  |
| Hypericaceae | *Hypericum campestre*Cham. & Schltdl. |  | x |  |
| Iridaceae | *Sisyrinchium chilense Hook.* |  | x |  |
|  | *Sysirinchium sp.* |  | x |  |
| Juncaceae | *Juncus sp.* |  | x |  |
|  | *Juncus tenuis* Willd. |  | x |  |
| Lamiaceae | *Hyptis mutabilis*(Rich.) Briq. | x | x |  |
|  | *Teucrium vesicarium*Mill. |  | x |  |
| Leguminosae | *Acacia caven*(Molina) Molina |  | x |  |
|  | *Aeschynomene histrix var. incana*(Vogel) Benth. | x |  |  |
|  | *Astragalus cf. distinens Macloskie* |  | x |  |
|  | *Chamaecrista repens*(Vogel) H.S.Irwin & Barneby | x |  |  |
|  | *Desmodium affine*Schltdl. |  | x |  |
|  | *Desmodium sp.* |  | x |  |
|  | *Desmanthus virgatus*(L.) Willd. |  | x |  |
|  | *Lotus corniculatus*L. |  | x |  |
|  | *Lupinus multiflorus*Desr. | x |  |  |
|  | *Macroptilium prostratum*(Benth.) Urb*.* |  | x |  |
|  | *Prosopis affinis*Spreng |  | x |  |
|  | *Rhynchosia senna*Hook*.* |  | x |  |
|  | *Senna scabriuscula*(Vogel) H.S.Irwin & Barneby | x |  |  |
|  | *Stylosanthes sp.* | x |  |  |
|  | *Tephrosia cinerea*(L.) Pers. | x |  |  |
|  | *Zornia trachycarpa*Vogel | x |  |  |
| Lythraceae | *Cuphea glutinosa*Cham. & Schltdl. |  | x |  |
|  | *Heimia salicifolia*(Kunth) Link |  | x |  |
|  | *Lythrum maritimum*Kunth |  | x |  |
| Malvaceae | *Ayenia sp.* | x | x |  |
|  | *Krapovickasia flavescens*(Cav.) Fryxell |  | x |  |
|  | *Modiolastrum malvifolium*(Griseb.) K. Schum. |  | x |  |
|  | *Pavonia glechomoides*A. St.-Hil*.* |  | x |  |
|  | *Sida cf vespertina* Ekman |  | x |  |
|  | *Sida ciliaris L.* |  | x |  |
|  | *Sida rhombifolia L.* |  | x |  |
|  | *Sida spinosa L.* |  | x |  |
|  | *Wissadula glechomifolia* Hassl. |  | x |  |
| Meliaceae | *Melia azedarach L.* |  | x |  |
| Molluginaceae | *Mollugo verticillata L.* |  | x |  |
| Moraceae | *Dorstenia brasiliensis*Lam. | x |  |  |
| Myrtaceae | *Blepharocalyx salicifolius*(Kunth) O.Berg |  | x |  |
|  | *Eugenia myrcianthes*Nied. |  | x |  |
| Onagraceae | *Oenothera indecora*Cambess | x | x |  |
|  | *Oenothera parodiana*Munz | x |  |  |
|  | *Oenothera sp.1* |  | x |  |
| Orchidaceae | *Brachystele sp.* |  | x |  |
| Orobanchaceae | *Agalinis genistifolia*(Cham. & Schltdl.) D'Arcy |  | x |  |
| Oxalidaceae | *Oxalis conorrihiza* Jacq. |  | x |  |
|  | *Oxalis sellowiana* Zucc. | x |  |  |
|  | *Oxalis sp.* | x | x |  |
| Passifloraceae | *Passiflora sp.* | x |  |  |
| Plantaginaceae | *Kickxia elatine*(L.) Dumort*.* |  | x |  |
|  | *Plantago australis Lam.* | x | x |  |
|  | *Plantago brasiliensis*Sims | x |  |  |
|  | *Plantago tomentosa*Lam. |  | x |  |
|  | *Stemodia verticillata*(Mill.) Hassl. |  | x |  |
|  | *Veronica persica Poir.* |  | x |  |
| Poaceae | *Andropogon lateralis*Nees | x |  |  |
|  | *Andropogon sp.* | x |  |  |
|  | *Aristida circinalis* Lindm. | x |  |  |
|  | *Aristida sp.* | x |  |  |
|  | *Axonopus sp.* | x |  |  |
|  | *Bothriochloa laguroides*(DC.) Herter | x |  |  |
|  | *Briza sp.* | x | x |  |
|  | *Briza sp.1* | x | x |  |
|  | *Briza sp.2* |  | x |  |
|  | *Briza sp.3* |  | x |  |
|  | *Bromus catharticus* Vahl | x |  |  |
|  | *Calamagrostis sp.* |  | x |  |
|  | *Chloris* *elata* Desv. |  | x |  |
|  | *Chloris sp.* |  | x |  |
|  | *Cynodon dactylon (L.) Pers.* | x | x |  |
|  | *Digitaria aequiglumis*(Hack. & Arechav.) Parodi |  | x |  |
|  | *Digitaria sacchariflora*(Raddi) Henrard |  | x |  |
|  | *Echinochloa colona*(L.) Link | x | x |  |
|  | *Echinochloa sp.* |  | x |  |
|  | *Eleusine indica*(L.) Gaertn. |  | x |  |
|  | *Elionurus muticus*(Spreng.) Kuntze | x |  |  |
|  | *Eragrostis sp* |  | x |  |
|  | *Eragrostis sp1* |  | x |  |
|  | *Eragrostis sp2* |  | x |  |
|  | *Eustachys paspaloides*(Vahl) Lanza & Mattei | x | x |  |
|  | *Lolium sp.* | x |  |  |
|  | *Melica brasiliana Ard.* | x |  |  |
|  | *Panicum sabulorum Lam.* | x | x |  |
|  | *Panicum sp.* | x | x |  |
|  | *Paspalum* *dilatatum* Poir. |  | x |  |
|  | *Paspalum* *plicatulum* Michx. | x |  |  |
|  | *Paspalum distichum L.* |  | x |  |
|  | *Paspalum sp.* |  | x |  |
|  | *Paspalum sp.1* |  | x |  |
|  | *Piptochaetium montevidense*(Spreng.) *Parodi* | x | x |  |
|  | *Piptochaetium ruprechtianum É.Desv.* |  | x |  |
|  | *Piptochaetium sp.* |  | x |  |
|  | *Piptochaetium stipoides*(Trin. & Rupr.) Hack. & Arechav. |  | x |  |
|  | *Rottboellia sp.* | x |  |  |
|  | *Schizachyrium sp.* | x | x |  |
|  | *Setaria parviflora*(Poir.) M.Kerguelen | x | x |  |
|  | *Setaria sp.* |  | x |  |
|  | *Sporobolus sp.* | x |  |  |
|  | *Steinchisma hians*(Elliott) Nash |  | x |  |
|  | *Stipa sp.* | x |  |  |
|  | *Stipa sp.1* | x | x |  |
|  | *Stipa sp.2* | x | x |  |
| Polygalaceae | *Monnina* *cuneata* A. St.-Hil. | x | x |  |
|  | *Polygala duarteana A. St.-Hil. & Moq.* | x | x |  |
| Portulacaceae | *Portulaca grandiflora Hook.* |  | x |  |
|  | *Portulaca sp.* |  | x |  |
| Primulaceae | *Anagallis arvensis L.* |  | x |  |
| Ranunculaceae | *Clematis campestris A.St.-Hil.* |  | x |  |
| Rosaceae | *Margyricarpus pinnatus*(Lam.) Kuntze | x |  |  |
| Rubiaceae | *Galium aparine* L. | x |  |  |
|  | *Galium richardianum*(Gillies ex Hook. & Arn.) Endl. ex Walp. |  | x |  |
|  | *Mitracarpus megapotamicus*(Spreng.) *Kuntze* | x |  |  |
|  | *Richardia brasiliensis Gomes* | x | x |  |
|  | *Richardia humistrata*(Cham. & Schltdl.) *Steud.* | x | x |  |
|  | *Richardia stellaris*(Cham. & Schltdl.) Steud. |  | x |  |
|  | *Spermacoce glabra Michx.* |  | x |  |
|  | *Spermacoce verticillata L.* | x | x |  |
| Smilacaceae | *Smilax campestris Griseb.* |  | x |  |
| Solanaceae | *Bouchetia anomala*(Miers) Britton & Rusby |  | x |  |
|  | *Nicotiana longiflora Cav.* |  | x |  |
|  | *Petunia integrifolia*(Hook.) Schinz & Thell*.* | x | x |  |
|  | *Physalis angulata* L. |  | x |  |
|  | *Solanum atropurpureum* Schrank |  | x |  |
|  | *Solanum granuloso-leprosum* Dunal |  | x |  |
|  | *Solanum reflexum* Schrank |  | x |  |
|  | *Solanum sisymbriifolium Lam* |  | x |  |
|  | *Solanum sp.* |  | x |  |
|  | *Solanum sp1* |  | x |  |
| Urticaceae | *Parietaria debilis* G.Forst. |  | x |  |
| Verbenaceae | *Glandularia subincana*Tronc*.* |  | x |  |
|  | *Lantana megapotamica*(Spreng.) Tronc. | x |  |  |
|  | *Lippia cf arechavaletae* Moldenke | x |  |  |
|  | *Verbena bonariensis L.* |  | x |  |
|  | *Verbena gracilescens*(Cham.) Herter |  | x |  |
|  | *Verbena rigida*Spreng. |  | x |  |
| Violaceae | *Hybanthus parviflorus*(L.f.) Baill. | x | x |  |

References

The Plant List (2013) The Plant list. A working list of all plant species. http://www.theplantlist.org/
